# Tooth fragment embedded in the lower lip for 10 months following dentoalveolar trauma: A case report with literature review

**DOI:** 10.4103/2321-3868.135652

**Published:** 2014-07-28

**Authors:** N. B. Nagaveni, K. V. Umashankara

**Affiliations:** 1 Department of Pedodontics and Preventive Dentistry, College of Dental Sciences, Davangere 577002, Karnataka, India; 2 Department of Oral and Maxillofacial Surgery, Bapuji Dental College and Hospital, Davangere, Karnataka, India

**Keywords:** Fractured incisor, lip laceration, tooth embedding

## Abstract

Traumatic injuries to maxillary anterior teeth are a common finding in children because of falls while playing. Sequelae of trauma to dental hard tissue include broken, lost, aspirated and swallowed teeth. One additional hazard is the embedding of fractured tooth fragments in the soft tissues, particularly in the lip. A 10-year-old male patient complained of pain in the lower lip. There was a history of trauma to the upper anterior tooth 10 months previously. Clinical examination showed scarring and discoloration over the lower lip, and the presence of a hard mass was felt on palpation. Intraoral examination revealed an Ellis and Davey class II fracture of number 11. A radiograph of the lip was taken, which showed a radiopaque structure similar to the shape of the missing tooth fragment. Under local anesthesia, the tooth fragment was removed successfully, and the class II fracture was restored with composite. Therefore, proper clinical and complete radiographic examination of both hard and soft tissues following dental trauma is essential to rule out such occurrences.

## Introduction

Traumatic injuries to the maxillary anterior teeth are a common finding in children because of falls while playing.[1] The reason for the increased vulnerability of the maxillary incisors to fracturing is because of the projection of anterior teeth and the short labial lip that does not adequately protect these teeth.[2] Therefore, broken, lost, swallowed, or aspirated teeth can be a hazard in dental and medical practice. One additional hazard is the embedding of fractured tooth fragments in the soft tissues,[1–23] particularly in the lip. It is commonly observed that dental traumas are usually associated with damage to the surrounding soft tissues, varying from bruises to deep lacerations. From numerous case reports, it has been observed that an impact force toward the incisors leads to fracture and causes a laceration of the soft tissues, particularly of the lips, and may lead to embedding of tooth fragments in the lip [Table 1]. Such tooth fragments, if undetected at the time of emergency treatment, may remain undiagnosed for longer periods and lead to infection and disfiguring fibrosis in addition to medicolegal complications.[4] The present article reports a case of scarring and foreign body reaction in the lip due to embedding of an occult tooth fragment for approximately 10 months after dentoalveolar trauma without patient awareness.

**Table:** 

Access this article online	
**Quick Response Code**:	**Website**: www.burnstrauma.com
	**DOI**: 10.4103/2321-3868.135652

**Table 1:** **Published literature regarding tooth fragments embedded in soft tissues following dentoalveolar trauma**

Author/Year	Patient age (Years)/Gender	Involved soft tissue	Fractured tooth	Type of fracture	Type of treatment	Duration of tooth embedding in the soft tissues
Snawder et al., 1979[5]	—	—	—	—	—	—
Hill and Picton, 1981[6]	—	Tongue	11	Uncomplicated crown fracture	—	—
McDonnell and McKiernan, 1986[7]	—	Tongue	—	—	—	—
Wadkar et al., 1986[4]	—	Lower lip	11	—	—	—
Clark and Jones, 1987[8]	—	—	—	—	—	—
Taran et al., 1994[9]	7/F	Lower lip	11	—	Surgical removal	Protruded 18 days later
Kalra and Aggarwal, 1997[10]	6/M	Upper lip	51	—	—	—
da Silva et al., 2005[11]	10/M	Lower lip	—	—	Surgical removal	—
	17/M	Lower lip				
Cetinkaya, 2005[12]	—	Lower lip	—	—	—	—
Pasini, 2006[13]	—	Lower lip	—	Uncomplicated crown fracture	Reattachment	—
Rao and Hegde, 2006[14]	9/M	Lower lip	—	—	Surgical removal	Spontaneous eruption 8 months after the trauma
Naudi and Fung, 2007[15]	—	Lower lip	—	Uncomplicated crown fracture	Reattachment	—
Schwengber et al., 2010[16]	8/M	Lower lip	21	—	Reattachment	—
Al-Jundi, 2010[17]	—	Lower lip	—	—	—	Noticed 18 months after the trauma
Cubukcu, 2011[18]	—	Upper lip	Primary central incisor	—	Surgical removal	—
Sangwan, 2011[19]	8/F	Lower lip	11	—	Reattachment	—
Antunes et al., 2012[20]	—	Lower lip	—	—	—	—
Lauritano et al., 2012[21]	10/M	Lower lip	21	—	Reattachment	—
Lips, 2012[3]	8/M	Lower lip	21	—	Reattachment	—
Goodson and Bhangoo 2013[21]	—	—	—	—	—	—
Agarwal et al., 2013[2]	—	Upper lip	—	—	—	—
Barua, et al. 2013[22]	12/F	Lower lip	—	—	Surgical removal	Present for 5 months in the lip
Altundasar and Demiralp, 2013[23]	23/M	Lower lip	11	—	Surgical removal	—
Present case	10/M	Lower lip	11	Uncomplicated crown fracture	Surgical removal	Present for 10 months in the lip

## Case Report

**Figure 1:**
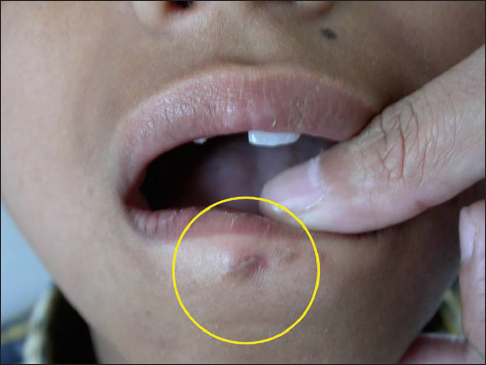
Photograph showing scarring and discoloration of the lower lip.

**Figure 2:**
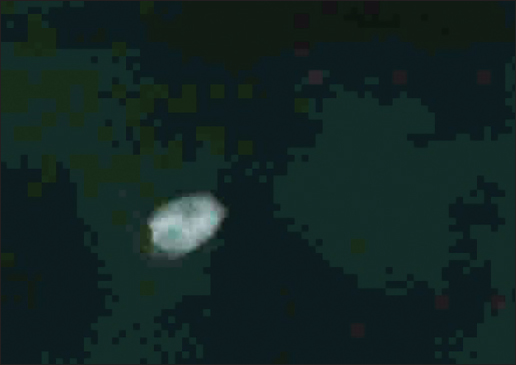
Radiograph of the lip showing the radiopaque tooth fragment.

**Figure 3:**
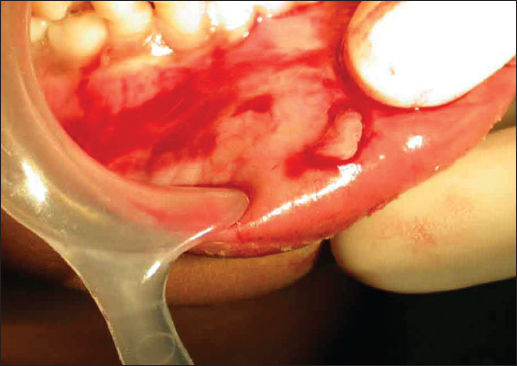
Exposure of the embedded tooth fragment.

**Figure 4:**
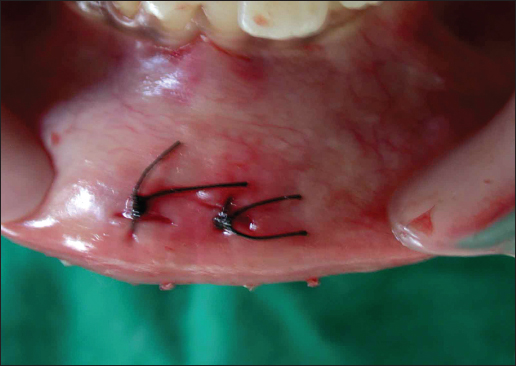
Postoperative photograph after suturing.

**Figure 5:**
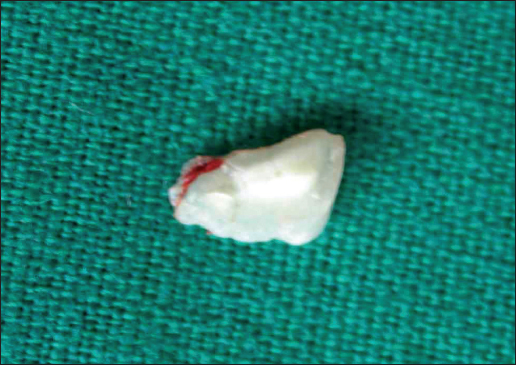
Photograph of the tooth fragment embedded in the lip.

A 10-year-old male patient reported to the department of Pedodontics complaining of pain in the lower lip that began 15 days before. The patient’s medical history revealed traumatic injury to the upper anterior teeth in addition to a lower lip laceration due to a fall while playing approximately 10 months previously. However, at the time, no attempt was made to locate the fractured tooth fragment at the site of the accident neither by the patient nor by his parents. The patient consulted a nearby general medical practitioner for the soft tissue injury, and the lip laceration was sutured by the medical practitioner. Subsequently, the patient did not receive any treatment for the fractured upper teeth. For approximately 10 months, the patient did not feel any discomfort in the lip. Fifteen days before presenting at the clinic, the patient began to have pain in the lower lip. Clinical examination showed scarring and discoloration of the skin to the right of the midline of the external aspect of the lip [Figure 1]. On palpation, a small hard mass was felt in the lip mucosa. Intraoral examination revealed an Ellis and Davey class II fracture involving the permanent maxillary right central incisor. Suspecting a foreign body, a radiograph of the lip was taken, which showed a radiopaque structure similar to that of the fractured tooth fragment [Figure 2]. Based on the patient’s history and on clinical and radiographic findings, the present case was diagnosed as a case of embedded fractured tooth fragment in the lip following trauma. The embedding of the tooth fragment in the lower lip was explained to the patient, and its removal under local anesthesia was planned. A small incision was made under local anesthesia in the proximity of the hard mass, which exposed the embedded tooth fragment [Figure 3]. The tooth fragment was completely removed followed by suturing with 3−0 black silk suture [Figure 4]. Examination of the tooth fragment [Figure 5] confirmed that it was a tooth fragment that had been embedded and undetected in the lip for almost 10 months. The patient was reviewed regularly, and healing was uneventful. The fractured tooth fragment was discarded, as the patient did not agree to the reattachment procedure. Later, the fractured upper anterior tooth was restored with composite.

## Discussion

Traumatic injury to the upper anterior teeth is most commonly encountered in the first decade of life, with falls being the most frequent etiology. It has been reported that these injuries occur in association with soft tissue lacerations; as a result, the embedding of the fractured tooth fragment in the surrounding soft tissues is a common sequela.[1–23] An extensive review of PubMed literature revealed reports of tooth fragments embedded in various soft tissues [Table 1].[1–23] Among these, in most of the reported cases (16 out of 24 case reports), the lower lip was the most common site for the embedded incisor fragment.[1,3,4,9,11–17,19,20,22,23] However, Kalra *et al.*,[10] Cubukcu *et al.*,[18] and Agarwal *et al.*[2] reported cases with tooth fragments embedded in the upper lip. McDonnell and McKiernan (in 1986)[7] and Hill *et al.*[6] published case reports of tooth fragments embedded in the tongue.

There are reports of spontaneous eruption of the undetected tooth fragments from the soft tissues. If the tooth does not erupt and remains within the soft tissues, persistent chronic infection with pus discharge and disfiguring fibrosis may occur. In 2010, Al-Jundi[17] reported a case of a tooth fragment embedded in the lower lip that remained undiagnosed for 18 months. This is the only report in the literature documenting the duration of a tooth embedded in the lip for this length of time. In 2006, Rao and Hegde[14] published a case report on the spontaneous eruption of the occult tooth after 8 months of entrapment in the lower lip. In the present case, the tooth remained within the lip for 10 months, leading to fibrosis and discoloration of the skin. A recent report published by Barua *et al.* [22] described a tooth fragment embedded in the lower lip for 5 months [Table 1].

Tooth fragments embedded in the soft tissue may not be easily detectable clinically. Therefore, every attempt should be made to locate the missing tooth structure before the wound is closed. If laceration and bleeding make clinical examination difficult, simple soft tissue and occlusal radiographs should be taken to help detect tooth fragments entrapped in the oral soft tissues.[2,18,23] Once the embedded tooth is diagnosed on radiographs, complete removal of the fractured tooth fragment is important to prevent infection, disfiguring, scarring and discoloration of the skin.

In the present case, it was noted that neither the patient nor the parents attempted to locate the tooth fragment at the site of injury. The medical practitioner who treated the patient also did not evaluate the possibility of embedded tooth fragments in the lacerated lip following the trauma. From this information, it is evident that there is a lack of knowledge among some health professionals regarding tooth embedding in soft tissues following trauma. Moreover, it has been reported that a majority of the skin wounds in children are often repaired in general medical hospitals. Additionally, it was found that the medical practitioners provided the treatment without detecting the foreign body or even seeking the opinion of a dental specialist (pediatric dentist) about the management of such injuries.[16,19] Therefore, collaboration between medical and dental professionals is essential in the management of such cases. Following retrieval of the embedded tooth fragment, the fragment can be used to restore the remaining fractured tooth. In the present case, the tooth fragment was discarded because the patient’s parents did not agree to the reattachment procedure. A literature search revealed 6 cases with reattachment procedures using the tooth retrieved from the soft tissues in addition to the long-term results.[1,3,13,15,16,19]

Finally, this paper emphasizes the importance of a detailed physical and radiographic evaluation of these patients following orofacial trauma. Particularly in cases of dental trauma that presents with soft tissue injuries such as lip laceration, both the hard tissue and the adjacent soft tissue should be carefully examined, even if the soft tissue has been sutured and treated by another health professional during the emergency care. Because of the magnitude of soft tissue trauma associated with a minor tooth structure, the pediatric dentist may often be the first health provider to see the child. Thus, the pediatric dentist should look for missed tooth fragments in a child with such injuries.

## Fearured Articles on Wound Healing and Tissue Engineering in Burns & Trauma

**Table:**

• Current progress of skin engineering: seed cells, bioscaffolds, and construction strategies	BURNS & TRAUMA 2013; 1:63–72
• Biofilm delays wound healing: A review of the evidence	BURNS & TRAUMA 2013; 1:5–12
• Mesenchymal stem cells in tissue repairing and regeneration: Progress and future	BURNS & TRAUMA 2013; 1:13–20
• Collective cell migration: Implications for wound healing and cancer invasion	BURNS & TRAUMA 2013; 1:21–6
• Burn blister fluids in the neovascularization stage of burn wound healing: A comparision between superficial and deep partial-thickness burn wounds	BURNS & TRAUMA 2013; 1:27–31
• Honey exposure stimulates wound repair of human dermal fibroblasts	BURNS & TRAUMA 2013; 1:32–8
• An electrospun scaffold loaded with anti-androgen receptor compound for accelerating wound healing	BURNS & TRAUMA 2013; 1:95–101
• Regulation challenge of tissue engineering and regenerative medicine in China	BURNS & TRAUMA 2013; 1:56–62
